# Estimating the power to detect a change caused by a vaccine from time series data

**DOI:** 10.12688/gatesopenres.13116.2

**Published:** 2020-10-19

**Authors:** Daniel M. Weinberger, Joshua L. Warren

**Affiliations:** 1 Department of Epidemiology of Microbial Diseases, Yale School of Public Health, New Haven, CT, 06520, USA; 2 Public Health Modeling Unit, Yale School of Public Health, New Haven, CT, 06520, USA; 3 Department of Biostatistics, Yale School of Public Health, New Haven, CT, 06520, USA

**Keywords:** time series analysis, program evaluation, vaccine evaluation, power calculation

## Abstract

When evaluating the effects of vaccination programs, it is common to estimate changes in rates of disease before and after vaccine introduction. There are a number of related approaches that attempt to adjust for trends unrelated to the vaccine and to detect changes that coincide with introduction. However, characteristics of the data can influence the ability to estimate such a change. These include, but are not limited to, the number of years of available data prior to vaccine introduction, the expected strength of the effect of the intervention, the strength of underlying secular trends, and the amount of unexplained variability in the data. Sources of unexplained variability include model misspecification, epidemics due to unidentified pathogens, and changes in ascertainment or coding practice among others. In this study, we present a simple simulation framework for estimating the power to detect a decline and the precision of these estimates. We use real-world data from a pre-vaccine period to generate simulated time series where the vaccine effect is specified *a priori*. We present an interactive web-based tool to implement this approach. We also demonstrate the use of this approach using observed data on pneumonia hospitalization from the states in Brazil from a period prior to introduction of pneumococcal vaccines to generate the simulated time series. We relate the power of the hypothesis tests to the number of cases per year and the amount of unexplained variability in the data and demonstrate how fewer years of data influence the results.

## Introduction

After a new vaccine is introduced, it is often necessary to evaluate the effect of the intervention on disease rates. This is typically done by evaluating changes in the average number of cases or the trend in cases before and after vaccine introduction ^1^. However, this type of analysis is challenging because it can be difficult to distinguish changes in disease rates caused by the vaccine from changes resulting from random variations, epidemics, changes in healthcare utilization, or changes in reporting practices. Additionally, there is often no ‘ground-truth’ against which estimates can be compared to determine whether an estimate is credible. These issues are a threat to the validity of any vaccine evaluation study, even when using large nationwide databases. However, the problems are especially acute when moving from larger to smaller populations, where the signal to noise ratio will be lower.

While it is intuitive that having more noise in the data makes it more difficult to detect a change, it is not clear how much data are needed to effectively quantify a vaccine-associated change if one exists. The power to detect a decline will depend on many factors, including the magnitude of the expected effect (higher power with a greater expected decline), the number of cases per unit time, and the number of years of pre- and post-vaccine data. Because the specific characteristics of datasets can vary, it is difficult to make general statements about power. However, simulation-based methods can be used to evaluate and compare power in different datasets based on the pre-vaccine time series and the magnitude of the expected effects ^2, 3^.

In this study, we present a simple web-based tool that can be used to input any disease time series and obtain an estimate of the power for that series to detect a specified vaccine-associated decline. This is accomplished by extracting characteristics of the time series (e.g., seasonality, trends, unexplained variability) from the pre-vaccine period and simulating a set of time series that have similar characteristics and have a vaccine impact that is specified *a priori*. We demonstrate the application of this approach on observed pre-vaccine data on pneumonia hospitalizations from the 26 states plus the federal district in Brazil and relate characteristics of the time series to the power.

## Methods

### Data

We used state-level hospitalization data from Brazil, which have been described in detail previously ^4^. These de-identified data are drawn from the Unified Health System (SIH-SUS, Ministry of Health), which captures ~70% of the population in Brazil. The raw data can be obtained directly by contacting the Ministry of Health in Brazil. The formatted time series data are available in the Github repository for this study. Each hospitalization is assigned a unique ICD10 code. For these analyses, we focused on data on <12 month old children and 80+ year old adults for the pre-vaccine period 2003–2009. These two populations provide a useful contrast. The time series for the infants was relatively stable prior to vaccine introduction, while the data for the 80+ year old adults had a notable increasing trend before vaccine introduction. Both sets of time series exhibit strong seasonality with a peak in the winter. 

### Simulating data based on observed time series from Brazil

The goal for this exercise was to simulate a set of time series with characteristics that resembled the observed hospitalization data from the pre-vaccine period but that had specified vaccine effects added in. We then sought to estimate the vaccine effect using the same model that was used to generate the data and evaluate the power to detect the effect. This provides a best-case scenario where the underlying model is correctly specified. 

The first step in this process was to extract characteristics of the time series from the pre-vaccine period (trend, seasonality, and amount of unexplained variation). For each state, we fit a regression model to the data from the pre-vaccine period (2003–2009). The outcome variable was the number of pneumonia hospitalizations (coded as J12-18) per month, and the covariates were an index variable for time (to capture any linear trends in the data) and 12-month and 6-month harmonic variables (to capture seasonality) ^5^. We used a Poisson regression model with a Gaussian observation-level random intercept to account for overdispersion in the data such that

$${\text{Y}}_{\text{t}}\;\sim \text{Poisson}(e^{\mu_{t}+\phi_{t}}),$$

$${\text{μ}}_{\text{t}}={\text{β}}_{0}+{\text{β}}_{1}*{\text{time}}_{\text{t}}+{\text{β}}_{2}*\text{cos}({\text{θ}}_{\text{t}}/12)+{\text{β}}_{3}*\text{cos}({\text{θ}}_{\text{t}}/12)+{\text{β}}_{4}*\text{sin}({\text{θ}}_{\text{t}}/6)+{\text{β}}_{5}*\text{sin}({\text{θ}}_{\text{t}}/6),\;\text{and}$$

$${\text{θ}}_{\text{t}}=2*\text{π}*{\text{time}}_{\text{t}}\text{.}$$

The models were fit using the glmer function in the lme4 package in R, version 3.6.1.

The next step is to use the fitted model to simulate time series of counts of hospitalization for the post-vaccine period with similar characteristics as the pre-vaccine period, with a specified vaccine-associated decline added to the simulated data. Using the estimated regression coefficients $\left({\hat{\text{β}}}_{\text{k}}\right)$ and their estimated variance/covariance matrix, we generated 500 independent random draws of the parameters from a multivariate normal distribution for each state and age group combination. These were combined with the design matrix to obtain simulated ${\hat{\mu }}_{t}.$ Random draws of ${\hat{\phi }}_{t}$ were independently generated from a normal distribution with a mean of 0 and a standard deviation equal to the standard deviation estimated for *ϕ _t_* from the fitted model. To incorporate a known vaccine effect, we assumed that the time series declined by a specified amount over a 24-month period. We generated time series where the maximum vaccine effect after 24 months ranged from a 10% – 50% reduction (rate ratio of 0.5–0.9). To capture these declines, we generated a vector, ****v****_t_, with entries equal to 0 at the time of vaccine introduction and decreasing linearly to the log(Rate-Ratio-Final) over 24 months. Simulated counts, *Y*_*t*,sim_, were generated by taking a random draw from the Poisson distribution with mean $e^{{\hat{\mu }}_{t}+{\hat{\phi }}_{t}+v_{t}}.$ The simulated counts reflect uncertainty in the regression parameters, unexplained variability in the data, as well as uncertainty from the observation process. The simulated counts from the post-vaccine period were combined with the observed counts from the pre-vaccine period. Therefore, the power calculations are conditional on the observed pre-vaccine data.

### Estimation of vaccine effects

We next estimated the vaccine effect using a regression model similar to the one used to generate the data. The outcome was the (simulated) number of counts per month. As above, we adjusted for seasonality using 6- and 12-month harmonic terms, and secular trends were captured using an index for time. The vaccine effect was quantified using a linear spline term that began at the time of vaccine introduction and continued for 24 months before stabilizing. An observation-level random intercept was included to capture overdispersion of the count data. Using the fitted model, we calculated the estimated rate ratio 24 months after vaccine introduction as 24*(coefficient for the vaccine effect term). To evaluate how many years of pre-vaccine data are needed to estimate the effects, we sequentially removed the first 1, 2, or 3 years of data and evaluated the effect on power. Coverage of the 95% confidence intervals (alpha=0.05) were used to assess power.

### Data and availability

All of the time series data and code used in these analyses are available from a Github repository https://github.com/weinbergerlab/PoissonITS_power. The interactive tool, along with a sample dataset, can be accessed at https://weinbergerlab.shinyapps.io/ITS_Poisson_Power.

## Results

### Interactive tool to estimate power

**Figure 1.**
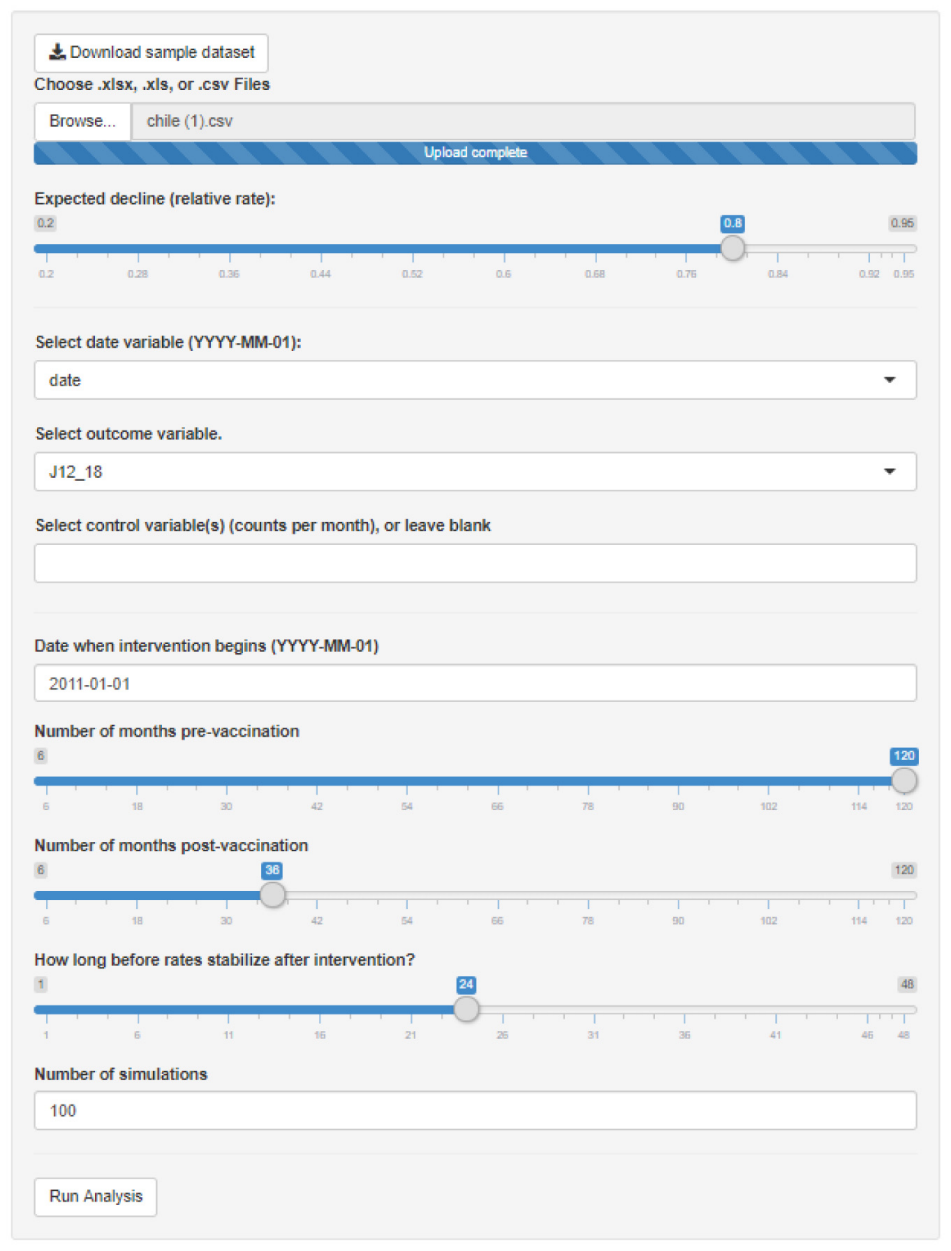
User interface for power calculation. The user uploads a time series, specifies the expected decline in terms of a rate ratio, specifies the key variables (date, outcome of interest, and controls), the date of the intervention, and the number of simulations to generate. A sample dataset can be downloaded by clicking the button at the top of the screen.

Because the power to detect a change in a time series is influenced by the expected effect size, the amount of unexplained variation in the data, and the number of years of data available, it can be difficult to make general statements about power. However, observed time series from the pre-vaccine period can be used to simulate time series to perform a best-case power calculation. This can provide an indication of whether it is worth performing an analysis or whether collecting additional data (e.g., additional pre-vaccine time points) could be helpful. We provide a simple ‘point-and-click’ interface where the user provides a time series in a csv or Excel format, indicates which columns contain the date variable, the outcome, and any potential controls, and the date at which the intervention is introduced ( Figure 1). Controls are time series that share important characteristics with the outcome time series. Relevant controls could include population size, all-cause hospitalizations, or other specific causes of disease that share similar trends and are not influenced by the intervention.

### Characteristics of the state-level data from Brazil

**Figure 2.**
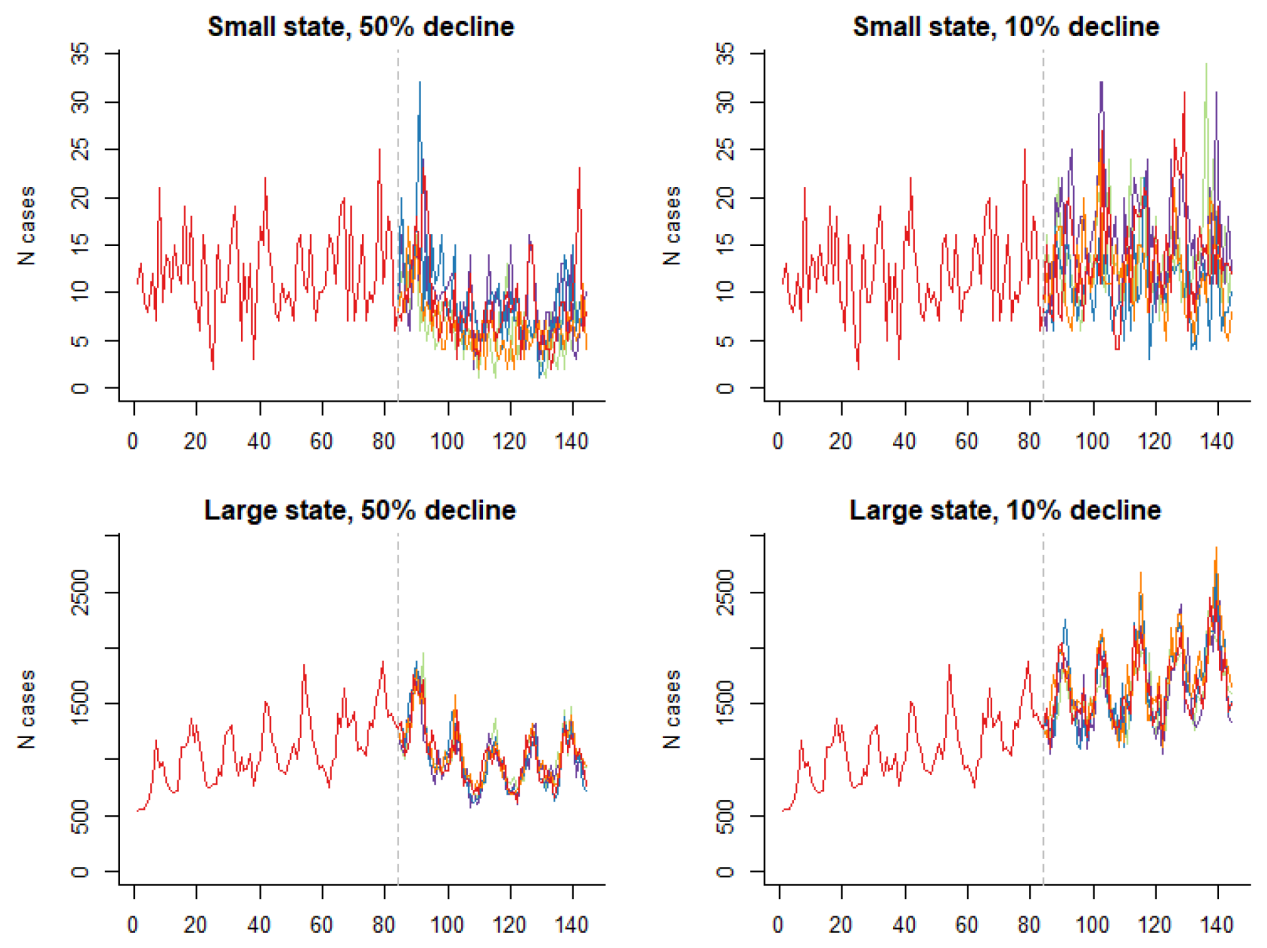
Sample simulated monthly time series of hospitalizations due to all-cause pneumonia for adults 80± years of age from a small state ( **A,B**) and a large state ( **C,D**) in Brazil with a 50% decline post-vaccination ( **A,C**) or 10% ( **B,D**).

As a demonstration of this approach, we apply this simulation framework to data from Brazil, disaggregated to different subnational levels (state, region). The size of the population varies drastically by state, from 450,000 to 41 million individuals (in 2010). On average there were 30-1900 hospitalizations due to pneumonia per month per state among children <12 m and 12-1100 hospitalizations per month per state among adults 80+ years of age during the pre-vaccine period. The time series for the <12m old children were highly seasonal but without a strong long-term trend, while the time series for the 80+ year olds increased markedly starting in the pre-vaccine period. We simulated time series for each of the states that had similar characteristics to the observed time series in the pre-vaccine period but with vaccine effects of different magnitudes ( Figure 2).

### Effect of number of cases and random noise on ability to accurately to detect a decline

**Figure 3.**
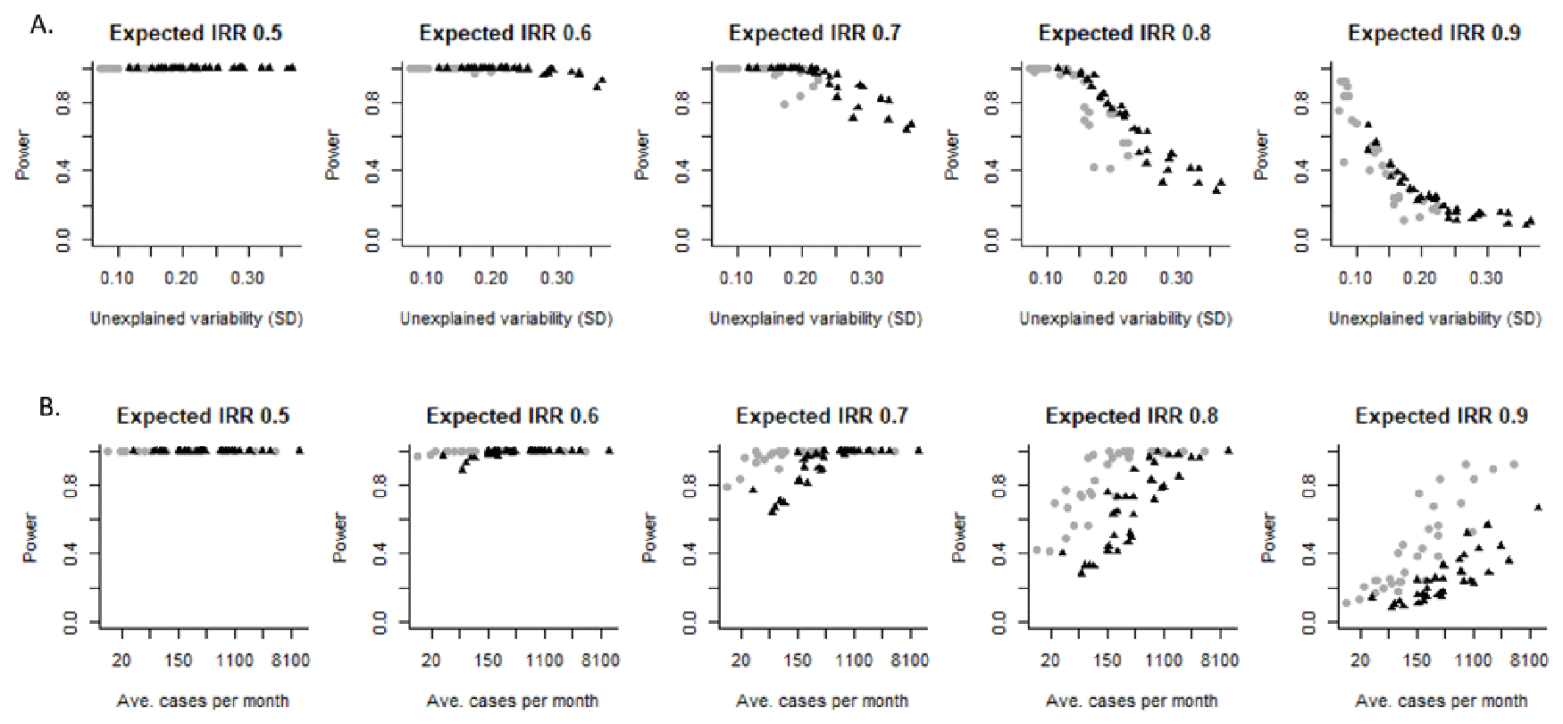
Relationship between power to detect a decline associated with vaccine introduction and ( **A**) the amount of unexplained variation in the time series or ( **B**) the average number of cases per month for different specified magnitudes of vaccine effects. The labels at the top of the panel indicate the magnitude of the expected vaccine effect, with an incidence rate ratio (IRR) of 0.5 representing a 50% decline associated with the vaccine and a IRR of 0.9 equal to a 10% decline. Each dot represents the power for one state in Brazil. The black triangles represent estimates for adults 80+ years of age, and the gray circles represent estimates for children <12 months of age.

We first evaluate the relationship between the amount of unexplained variability in the data and the ability to accurately estimate the effect of the vaccine. There is a clear relationship between the amount of unexplained variability in the data and the power to detect a vaccine-associated change ( Figure 3A). This trend was consistent across all of the states between both children and adults.

Plotting the estimated power against the average number of hospitalizations in the state/region, there is also a relationship, but the trend differed between children and adults ( Figure 3B). This is because the amount of unexplained variability was higher in the <12m old children than in the 80+ year old adults ( *Extended data:* Figure S1)

### Effect of number of years of data on ability to accurately to detect a decline

**Figure 4.**
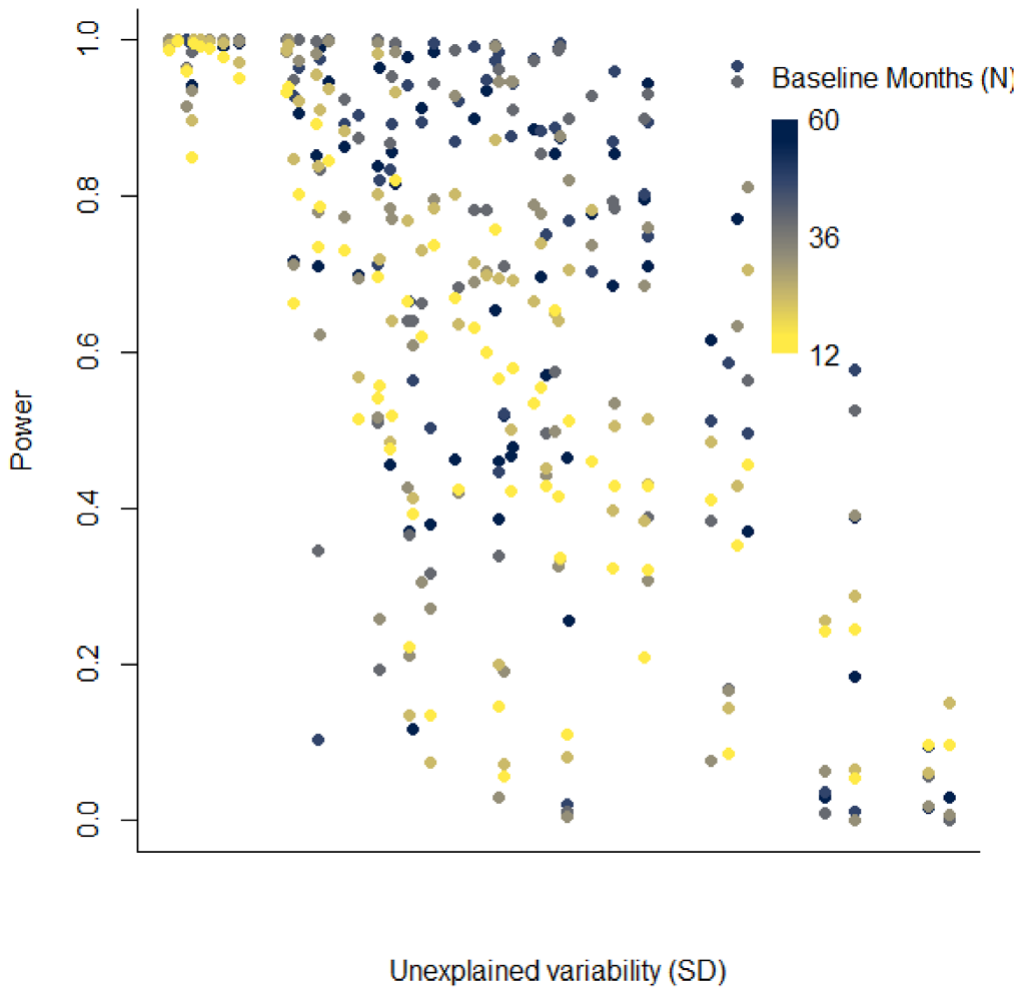
Effect of shortened baseline periods on the relationship between the power to detect a decline associated with vaccine introduction and the amount of unexplained variation in the time series. Each dot represents the power for one state/age group in Brazil. Dots with lighter colors had fewer years of data.

With fewer years of baseline data, the power to detect a change in disease rates associated with the vaccine also declines. For datasets with little unexplained variability, even with just 12 months of pre-vaccine data, there could be high power to detect a vaccine-associated decline of 20%. However, when there is more unexplained variability in the time series, power declines with shorter pre-vaccine periods ( Figure 4). These declines in power are particularly dramatic for time series with intermediate levels of unexplained variability ( Figure 4).

### Demonstration of the interface

**Figure 5.**
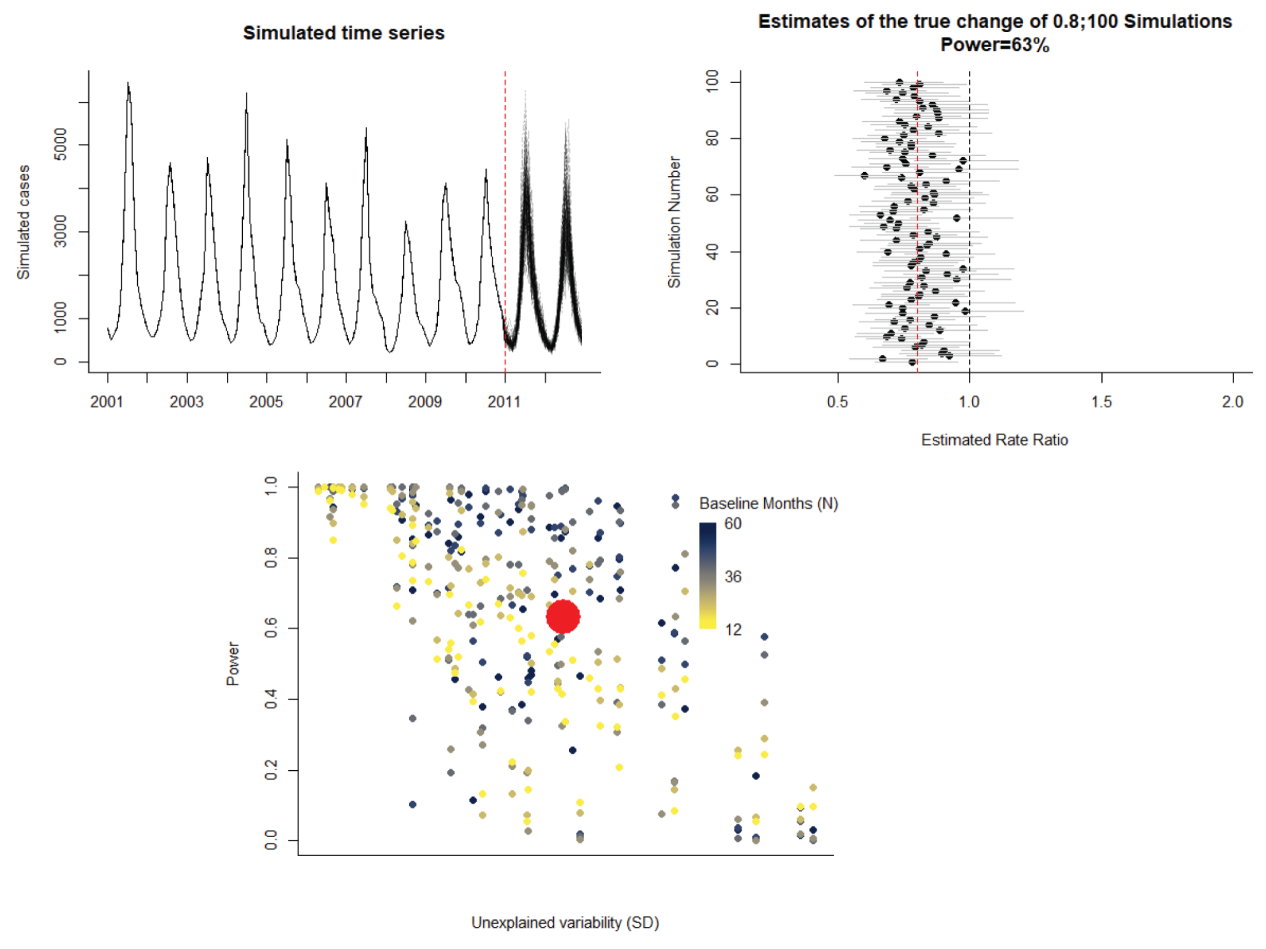
Sample output from the interactive interface using hospitalization data for Chile under 2 years of age (data can be obtained by clicking the download button on the interface). The upper left panel shows the 100 simulated time series. The upper right panel shows the estimates of the rate ratio for each of the 100 simulations. The true specified rate ratio (0.8) is denoted by a red dashed line. 63% of the estimates had 95% confidence intervals that did not cross 1. The bottom left panel shows the estimate of power for this study (red dot) compared with the estimates from the Brazil states with different length baseline periods.

As a demonstration of the point-and-click interface, we use hospitalization data from Chile among children <24 months (raw data available from http://www.deis.cl/) ^6^. This sample time series can be downloaded directly from the interface. The outcome variable is the number of hospitalizations per month due to all-cause pneumonia (J12_18) for 2003–2014. The number of non-respiratory hospitalizations per month (ach_noj) is included as a control time series. If no control is present, this field can be left blank. The date of vaccine introduction is set to January 1, 2011. The program generates a specified number of simulated post-vaccine time series (N) based on the pre-intervention data ( Figure 5A). With two years of post-vaccine data (vaccine introduction in 2011, evaluating through 2012), the 500 estimates of the rate ratio are centered on the true value (indicated by a red dashed line), with a moderate degree of uncertainty ( Figure 5B). This yields 63% power to detect a rate ratio of 0.8 ( Figure 5B). Compared with the analyses of the Brazil series, the power is reasonable given the amount of unexplained variability in the data but could be increased ( Figure 5C). This can be seen in the simulation by increasing the length of the evaluation period by a year (e.g., through 2013) (Extended data: Figure S2). The power in this instance would increase from 63% to 83% with an improvement in the precision of the estimates.

## Discussion

In this study, we describe a simple interface for conducting simulations to evaluate the power to detect a vaccine-associated decline from time series data. This approach provides analysts a simple best-case scenario for determining whether they are likely to detect specified vaccine effects with the data on hand or whether collecting additional pre- or post- vaccine data would be beneficial. This type of tool should be used when planning analyses and prior to conducting a formal evaluation analysis with the data on hand.

By analyzing subnational data from Brazil, we demonstrate how power varies with the number of cases and the degree of unexplained variability in the data. Reducing unexplained variability in the data by using time-varying covariates can help to increase power. Such covariate could include other causes of disease/hospitalization/death or known correlates of changes in disease rates (e.g., percent of the population with access to healthcare).

These analyses evaluate power based on the statistical characteristics of the time series. As with any analysis, failure to correctly control for relevant trends will also introduce important biases and could greatly outweigh the issues related to statistical characteristics of the data. For instance, if there is a non-linear trend that is not well-captured by an interrupted time series analysis, the vaccine effect could be substantially over- or under-estimated.

The estimates generated with this approach represent a ‘best-case’ scenario where we know the exact date of vaccine introduction and where all non-vaccine-associated changes are linear and can be controlled with a simple model. In reality, numerous factors can influence pneumonia hospitalization rates. The use of control variables can help to adjust for these, but often remain unexplained factors that cannot be easily adjusted.

In the statistical model used here, the control variable(s) are included as covariates in the regression framework. An alternative approach would be to pick a single covariate and include it as an offset term ^7^. There are advantages and disadvantages to using the control variables as covariates rather than an offset term (which effectively includes the control variable as a covariate but fixes the regression coefficient to one). Error in the control time series can lead to inadequate control of underlying trends ^8^. This is particularly an issue with data from small regions with few cases of disease. Using a control as an offset ensures that the trend is captured. However, using the control variable as an offset term can introduce biases when the control itself experiences major changes that are caused by a factor that does not reflect the outcome. By allowing the regression coefficient to be estimated instead of fixed at one, it guards against this by giving less weight to controls that do not reflect changes in the outcome.

In our analyses here and for the online tool, we use independent observation-level random effects. When analyzing time series data, there is often correlation between observations across time. Adjusting for seasonality and time-varying covariates can reduce this autocorrelation considerably in many settings, though residual autocorrelation can persist. An alternative approach would be to use an autoregressive model for the random effects. We have found that the autoregressive model of order one (AR(1)) for the random effects can lead to identifiability issues (similar to issues observed with spatial models ^9^) where introduction of the correlated random effects biases the estimation of trend or control variables. Depending on the characteristics of the underlying trends in the time series, this can bias estimates of vaccine impact.

The analyses presented here assume a gradual decline in disease rates following an intervention, which plateaus after 24 months. This would be a reasonable assumption for a new vaccine being introduced for routine use. In the case of a vaccine campaign, the change might be much more abrupt and resemble a step change. The interactive app allows the user to specify the length of time that passes before disease rates stabilize.

We summarize the results of these simulations in terms of statistical power (i.e., what percentage of simulations yielded a statistically significant effect when an actual non-zero effect was present). In practice, we typically avoid describing evaluations of vaccine impact made using observational time series data in terms of statistical significance. It is often more informative to instead describe the estimate of vaccine impact and the strength of the evidence/precision of the estimates. These types of analyses are rarely used for making dichotomous policy decision (e.g., licensure), so using an arbitrary threshold for declaring whether a vaccine ‘works’ is not needed.

In conclusion, we present a simple framework for evaluating the power to detect vaccine-associated declines of a specified magnitude. This approach can help in planning for an evaluation study and for understanding differences between studies.

## Data availability

### Underlying data

The Brazilian dataset can be accessed by contacting the Ministry of Health (Ministério da Saúde) directly via http://portalms.saude.gov.br.

The Chilean dataset can be accessed from the Chilean Department of Statistics website http://www.deis.cl Time series data and code available from: https://github.com/weinbergerlab/PoissonITS_power

Archived data and code as at time of publication: https://github.com/weinbergerlab/PoissonITS_power

License: CC0

### Extended data

Figshare: Estimating the power to detect a change caused by a vaccine from time series data,

This project contains the following extended data:

- **Figure S1.** Relationship between amount of unexplained variability in the data and the average number of cases per month for each of the Brazilian states for children <12 months of age (gray circles) and adults 80+ years of age (black triangles). https://doi.org/10.6084/m9.figshare.11908143 ^10^
- **Figure S2.** Sample output from the interactive interface using hospitalization data for Chile under 2 years of age, where the date of introduction is shifted earlier by 12 months (to January 1, 2010). In comparison to Figure 4, (where a date of introduction of January 1, 2011 is used), the estimates are more precise, and the power is higher. https://doi.org/10.6084/m9.figshare.11908158.v2 ^11^

Data are available under the terms of the Creative Commons Attribution 4.0 International license (CC-BY 4.0).

## Software availability

Interactive tool available from: https://weinbergerlab.shinyapps.io/ITS_Poisson_Power

Source code available from: https://github.com/weinbergerlab/PoissonITS_power

Archived source code as at time of publication: https://zenodo.org/badge/latestdoi/177850456

License: CC-BY 4.0
