# Supplementary material for: Estimating the power to detect a change caused by a vaccine from time series data
Source: Gates Open Res. 2020 Oct 19;4:27. Originally published 2020 Mar 9. [Version 2] doi: 10.12688/gatesopenres.13116.2 (PMC7578561; doi:10.12688/gatesopenres.13116.2)
Supplement: Peer review history

# Peer review: Reviewer response for version 2

**DOI:** 10.21956/gatesopenres.14384.r29849

**Published:** 2020-10-21

**Authors:** Christian Bottomley

**Affiliations:** 1 Department of Infectious Disease Epidemiology, London School of Hygiene and Tropical Medicine, London, UK

The authors have provided a thorough and thoughful reponse to my comments. The revised web-based tool seems to work well on the example dataset and I look forward to using it to plan future studies.

Is the work clearly and accurately presented and does it cite the current literature?

Yes

If applicable, is the statistical analysis and its interpretation appropriate?

Partly

Are all the source data underlying the results available to ensure full reproducibility?

Yes

Is the study design appropriate and is the work technically sound?

Partly

Are the conclusions drawn adequately supported by the results?

Yes

Are sufficient details of methods and analysis provided to allow replication by others?

Partly

Reviewer Expertise:

NA

I confirm that I have read this submission and believe that I have an appropriate level of expertise to confirm that it is of an acceptable scientific standard.

# Peer review: Reviewer response for version 1

**DOI:** 10.21956/gatesopenres.14294.r28669

**Published:** 2020-04-14

**Authors:** Corinne Levy, Naim Ouldali

**Affiliations:** 1 ACTIV, Saint-Maur-des-Fossés, France; 2 Unité d’épidémiologie clinique, Assistance Publique-Hôpitaux de Paris, Hôpital Robert Debré, Paris, France; 3 ACTIV (Pediatric Clinical and Therapeutical Association of the Val de Marne), Saint-Maur-des-Fossés, France; 4 Université Paris Est, IMRB-GRC GEMINI, Créteil, France

In this relevant paper, the authors try to estimate the power to detect a change (a decline) caused by a vaccine from time series data. To implement this approach, they created an interactive web-based tool. They used data on pneumonia from Brazil before pneumococcal conjugate vaccine introduction to generate the simulated time series and to demonstrate the use of this approach.

 Interestingly, whether in times series analysis or in before after studies, the specific question of power is rarely raised and from our point of view, this is the strength of this work. This relevant information can have an added value to perform impact studies. Although the results the authors presented were expected, such as increasing power when increasing years of surveillance before the intervention, the interest of this work is to provide the demonstration of this effect, and to allow quantification of this power.

 For administrative data bases, we understand the usefulness of this tool. However, many surveillance systems are specifically set up to prospectively follow the evolution of an outcome after vaccine implementation, and adding a posteriori several years of surveillance before the vaccine to improve power when analyzing data is clearly not possible. With the proposed interactive tool, the dataset has to be downloaded to perform the power analysis, meaning that the study is already finished.

 But of course, the major interest of a power calculation lies in the fact that it is calculated a priori and not after the data were collected. Thus, we were not convinced by: “This type of tool should be used when planning analyses and prior to conducting a formal evaluation analysis with the data on hand.” nor by “This approach can help in planning for an evaluation study and for understanding differences between studies.”

 Several revisions should be made to strengthen the current paper:

- First of all, the authors should add limitations of their study.
- To allow a priori power comutation, one suggestion would be the possibility of adding a module to simulate data based on expected population sizes with assumed variability based on previous studies and a defined number of years. This would allow adjusting the number of baseline years needed before the intervention and would be really helpful to appropriately assess public health intervention impact.
- Another model than Poisson regression could also be proposed such as ARIMA or negative binomial regression modelling.
- How was the adjustment made on the control outcome?
- Inclusion of an ARMA term in the model should also be considered, adjusting for seasonality may not be always sufficient to capture all the autocorrelation of the data
- The evaluation of the residuals, particularly with correlograms, is also an important point
- The authors should improve the clarity of their R scripts and should add explanatory text so that non-experts can better understand the model building.

Is the work clearly and accurately presented and does it cite the current literature?

Yes

If applicable, is the statistical analysis and its interpretation appropriate?

Partly

Are all the source data underlying the results available to ensure full reproducibility?

Partly

Is the study design appropriate and is the work technically sound?

Yes

Are the conclusions drawn adequately supported by the results?

Partly

Are sufficient details of methods and analysis provided to allow replication by others?

Partly

Reviewer Expertise:

Pediatric infectious disease epidemiology

We confirm that we have read this submission and believe that we have an appropriate level of expertise to confirm that it is of an acceptable scientific standard.

# Peer review: Reviewer response for version 1

**DOI:** 10.21956/gatesopenres.14294.r28685

**Published:** 2020-03-27

**Authors:** Christian Bottomley

**Affiliations:** 1 Department of Infectious Disease Epidemiology, London School of Hygiene and Tropical Medicine, London, UK

In their paper Weinberger and Warren describe a simulation-based approach for calculating statistical power in time series studies of vaccine impact. They apply their methodology to data from Brazil and Chile, using the latter to demonstrate a web-based interactive tool they have developed.

 I agree with the premise of the paper that power calculations may have an important role to play in designing vaccine impact studies, particularly in deciding how much pre and post vaccine data needs to be collected. And the web-based tool is potentially a useful tool for researchers planning a vaccine impact study.

 However, my major concern is that in its current form the tool has limited flexibility to evaluate power for different amounts of pre and post vaccine data. For example, if a researcher wants to know the power associated with different lengths of post-vaccine surveillance, given they have, say, three years of pre-vaccine surveillance, can they use the tool to calculate power for these scenarios? Based on my understanding of the Chile example, the amount of post-intervention data can only be increased if the pre-intervention data is reduced. This is a significant constraint since it is hard to imagine why a researcher would not want to use all the pre-vaccine data available.

 My recommendation would therefore be to adapt the current version of this tool to include numbers of pre and post vaccine observations as additional parameters.  In theory this should be straightforward to do by:

  

1. Fitting the model to pre-vaccine data
2. Using the resulting parameter estimates, and an assumed vaccine impact, to simulate additional pre-vaccine and post-vaccine data
3. Estimating vaccine impact based on the combined (observed + simulated) data
4. Using vaccine impact estimates from multiple simulations to estimate power.  

I have proposed combining observed and simulated data so that the estimated power is conditional on the observed data. An alternative approach would be to simulate all the pre-vaccine data to give an unconditional estimate, but I think this estimate is probably less useful given that the final analysis will almost certainly make use of the pre-vaccine data used in the power calculation.

 Some additional observations: 

1. In equation 3, the second harmonic is written in terms of 2 sine functions. I think this is a typo and one of these functions should be a cosine. Also, a reference for harmonic regression (e.g. chapter 5.4 in Forecasting Principles and Practice by Hyndman & Athanasopoulos) might be useful.
2. The tool allows for the inclusion of a control series. This is a nice feature, but it wasn’t clear to me how the relationship between the two time series is specified? Is the control included as a covariate in the regression model? In a recent paper (Bottomley et al. 2019) we argue that because the control is effectively trend + error it should be included as an offset term, otherwise confounding due to trend is not adequately accounted for. 
3. An important assumption of the model is that the observations are independent. This is a big assumption because time series are often autocorrelated. One way to relax the assumption would be to model the random effect (phi_t) as an autoregressive process. This might make fitting the model more complicated so I can see that you might not want to go down this route, but at a minimum the issue of autocorrelation should be mentioned in the discussion.
4. Statistical power must be defined with respect to a particular significance level. I presume the calculations are based on a 5% significance level (2-sided test), but I didn’t see this stated anywhere.
5. The model assumes a linearly increasing intervention effect on the log scale.  This seems reasonable for a vaccine that is introduced into a childhood vaccine programme without a catchup campaign. But what about vaccines that are introduced with a catchup campaign? A step function might be more appropriate for this scenario. The assumed mode of introduction should be mentioned in the discussion or the tool could be modified to allow for different modes of introduction.

Some typos in the web interface:

1. In the section “Results”, delete “N” from the sentence “The second plot shows the N estimate”.
2. In the section “Sample Data” missing “r” in children “These time series represent the number of cases among childen <24 months of age”.

Is the work clearly and accurately presented and does it cite the current literature?

Yes

If applicable, is the statistical analysis and its interpretation appropriate?

Partly

Are all the source data underlying the results available to ensure full reproducibility?

Yes

Is the study design appropriate and is the work technically sound?

Partly

Are the conclusions drawn adequately supported by the results?

Yes

Are sufficient details of methods and analysis provided to allow replication by others?

Partly

Reviewer Expertise:

NA

I confirm that I have read this submission and believe that I have an appropriate level of expertise to confirm that it is of an acceptable scientific standard, however I have significant reservations, as outlined above.

## References

[1] : Forecasting: principles and practice. *OTexts*.2018; Reference source

[2] : Analysing Interrupted Time Series with a Control. *Epidemiologic Methods*.2019;8(1) : 10.1515/em-2018-0010 10.1515/em-2018-0010

# Response

**Published:** 2020-08-26

**Authors:** Daniel Weinberger

We thank Dr. Bottomley for the constructive feedback on the analyses. We have responded by making all analyses conditional on the observed pre-vaccine data. Additionally, we have modified the user interface so that the user can modify the number of pre- and post-vaccine time points used when estimating power.

*Reviewer #1*

**

*In their paper Weinberger and Warren describe a simulation-based approach for calculating statistical power in time series studies of vaccine impact. They apply their methodology to data from Brazil and Chile, using the latter to demonstrate a web-based interactive tool they have developed.*

**

*I agree with the premise of the paper that power calculations may have an important role to play in designing vaccine impact studies, particularly in deciding how much pre and post vaccine data needs to be collected. And the web-based tool is potentially a useful tool for researchers planning a vaccine impact study.*

**

*However, my major concern is that in its current form the tool has limited flexibility to evaluate power for different amounts of pre and post vaccine data. For example, if a researcher wants to know the power associated with different lengths of post-vaccine surveillance, given they have, say, three years of pre-vaccine surveillance, can they use the tool to calculate power for these scenarios? Based on my understanding of the Chile example, the amount of post-intervention data can only be increased if the pre-intervention data is reduced. This is a significant constraint since it is hard to imagine why a researcher would not want to use all the pre-vaccine data available.*

*My recommendation would therefore be to adapt the current version of this tool to include numbers of pre and post vaccine observations as additional parameters.  In theory this should be straightforward to do by:*

1. *Fitting the model to pre-vaccine data*
*  *
2. *Using the resulting parameter estimates, and an assumed vaccine impact, to simulate additional pre-vaccine and post-vaccine data*
*  *
3. *Estimating vaccine impact based on the combined (observed + simulated) data*
*  *
4. *Using vaccine impact estimates from multiple simulations to estimate power.  *

*I have proposed combining observed and simulated data so that the estimated power is conditional on the observed data. An alternative approach would be to simulate all the pre-vaccine data to give an unconditional estimate, but I think this estimate is probably less useful given that the final analysis will almost certainly make use of the pre-vaccine data used in the power calculation.*

****

****

**This is an excellent suggestion, and we have implemented the suggested change in the Shiny App, and we have also updated the analyses in the main manuscript to be conditional on the observed pre-vaccine data. The user can now specify the number of months of post-intervention data (and pre-intervention data) using sliders, and then power is conditional on the observed data from the pre-vaccine period. The updated tool is available at https://weinbergerlab.shinyapps.io/ITS_Poisson_Power**

****

****

*Some additional observations: *

1. *In equation 3, the second harmonic is written in terms of 2 sine functions. I think this is a typo and one of these functions should be a cosine. Also, a reference for harmonic regression (e.g. chapter 5.4 in Forecasting Principles and Practice by Hyndman & Athanasopoulos) might be useful.*

**We have fixed this typo and added the reference, as suggested.**


** **

1. *The tool allows for the inclusion of a control series. This is a nice feature, but it wasn’t clear to me how the relationship between the two time series is specified? Is the control included as a covariate in the regression model? In a recent paper (Bottomley et al. 2019) we argue that because the control is effectively trend + error it should be included as an offset term, otherwise confounding due to trend is not adequately accounted for. *

**Indeed, the log(control+0.5) is included as a covariate, and the user can specify multiple control variables if available. We agree that error in the control time series can lead to inadequate control of underlying trends (see Shioda et al., Epidemiology 2019). In our experience with administrative data, using a control as an offset can introduce biases when the control itself experiences major changes that are caused by a factor that does not reflect the outcome. By allowing the regression coefficient to be estimated instead of fixed at one, it guards against this effect by downweighting controls that do not reflect changes in the outcome. This is an interesting point though for future methodological work to compare these approaches, and we have added a comment on this to the discussion.  **

1. *An important assumption of the model is that the observations are independent. This is a big assumption because time series are often autocorrelated. One way to relax the assumption would be to model the random effect (phi_t) as an autoregressive process. This might make fitting the model more complicated so I can see that you might not want to go down this route, but at a minimum the issue of autocorrelation should be mentioned in the discussion.*

**We agree. However, there are two reasons we prefer to use an iid random effect. First, in many instances, after adjusting for seasonality and control time series, the residual autocorrelation is negligible. Second, we have found that AR(1) models can suffer from identifiability issues where inclusion of the correlated random effect biases the estimates of trend or control variables. A similar phenomenon has been described for spatial data: (Hodges JS, Reich BJ. Adding spatially-correlated errors can mess up the fixed effect you love. The American Statistician 2010; 64:325-34). Depending on the characteristics of the underlying trends in the time series, this can bias estimates of vaccine impact. We have not yet found a satisfactory solution to this problem, nor have we been able to determine the time series characteristics that lead to this identifiability issue. This is an area we are actively investigating. We have added a comment to the discussion to indicate that the use of an AR(1) random effect could lead to bias in the primary associations of interest.**


** **

1. *Statistical power must be defined with respect to a particular significance level. I presume the calculations are based on a 5% significance level (2-sided test), but I didn’t see this stated anywhere.*

**We have an alpha of 0.05 (2 sided). We have added this detail to the methods section.**


** **

1. *The model assumes a linearly increasing intervention effect on the log scale. This seems reasonable for a vaccine that is introduced into a childhood vaccine programme without a catchup campaign. But what about vaccines that are introduced with a catchup campaign? A step function might be more appropriate for this scenario. The assumed mode of introduction should be mentioned in the discussion or the tool could be modified to allow for different modes of introduction.*

**We agree and have added a comment on this to the discussion. We also modified the app to allow the user to specify the length of time over which the decline occurs. So for a step change, the length of time would be set to 1.**

**Some typos in the web interface:**

1. *In the section “Results”, delete “N” from the sentence “The second plot shows the N estimate”.*
*  *
2. *In the section “Sample Data” missing “r” in children “These time series represent the number of cases among childen <24 months of age”.*

**Thank you, we have fixed these typos.**
